# Ex vivo lung perfusion in donation after circulatory death lung transplantation: A systematic review and meta-analysis

**DOI:** 10.1016/j.jhlto.2026.100480

**Published:** 2026-01-04

**Authors:** Bianca S. Costa, Bruna C. Coelho, Iago T.C. Grillo, Rachid Eduardo Noleto da Nobrega Oliveira, Tulio Caldonazo, Felipe S. Passos, Erlon de Avila Carvalho

**Affiliations:** a Department of Medicine, University Nine of July, Bauru, Brazil; b Department of Medicine, Federal University of Ouro Preto, Ouro Preto, Brazil; c Department of Medicine, University of Buenos Aires, Buenos Aires, Argentina; d Department of Thoracic Surgery, Barretos Cancer Hospital, Barretos, Brazil; e Department of Cardiothoracic Surgery, Jena University Hospital, Jena, Germany; f Department of Thoracic Surgery, Mater Dei Hospital, Salvador, Brazil; g Department of Thoracic Surgery, UFMG Clinical Hospital, Belo Horizonte, Brazil

**Keywords:** Lung transplantation, Donation after circulatory death, EVLP, Organ preservation, Marginal donors

## Abstract

**Background:**

Lung transplantation is the definitive therapy for end-stage lung disease, but donor shortages contribute to high waiting list mortality. Donation after circulatory death (DCD) expands the donor pool, and ex vivo lung perfusion (EVLP) enables graft assessment and optimization. However, its impact in this setting remains uncertain. This meta-analysis aimed to evaluate whether EVLP in DCD lungs affects graft function and short-term outcomes compared with direct transplantation.

**Methods:**

Three databases were searched. The main outcome was grade 3 primary graft dysfunction (PGD). Additional outcomes included intensive care unit (ICU) and hospital length of stay (LOS), both analyzed quantitatively. Outcomes assessed qualitatively comprised short-term survival, pneumonia, and acute rejection. Random-effects models were applied to quantitative analyses.

**Results:**

Five observational studies (654 patients) were included. The incidence of grade 3 PGD was comparable between EVLP and non-EVLP groups (RR 1.29; 95%CI 0.97 to 1.71; *p* = 0.08; *I*² = 12.63%). Similarly, ICU LOS (*p* = 0.12) and hospital LOS (*p* = 0.83) were also comparable. Qualitative assessment showed no apparent differences in short- and mid-term survival or in the frequency of pneumonia and acute rejection between groups.

**Conclusion:**

EVLP in DCD lung transplantation was not associated with significant differences in grade 3 PGD, ICU or hospital LOS, or short- to mid-term outcomes compared with direct transplantation. Given its time and resource demands, EVLP may not be necessary for all DCD grafts; however, it remains particularly valuable for evaluating uncertain-quality lungs, where its selective use can help ensure graft safety.

## Background

Lung transplantation is currently the most effective therapy for patients with end-stage lung disease. However, organ shortage represents a significant limitation in several countries, resulting in high mortality rates on waiting lists, which can reach 20%. Accordingly, there is growing interest in the use of lungs from donation after circulatory death (DCD), a strategy to expand the donor pool, with the potential to increase transplant activity by nearly 28%.^1^, ^2^, ^3^, ^4^

Most lung transplants are still performed using organs from donors after brain death (DBD), which present higher utilization rates. In contrast, DCD grafts raise concerns related to ischemia time, logistical challenges, and the lack of standardized protocols. Despite these limitations, observational studies have shown similar clinical outcomes between the groups, including survival and incidence of primary graft dysfunction (PGD).^5^, ^6^, ^7^, ^8^, ^9^, ^10^

To increase the use of DCD lungs, ex vivo lung perfusion (EVLP) has been increasingly adopted. Although not required for all DCD donors, EVLP is generally reserved for marginal or borderline grafts, such as those with a prolonged agonal phase or suboptimal oxygenation. By preserving the lung in a normothermic circuit with a membrane oxygenator for up to 6 hours, EVLP enables continuous assessment of respiratory mechanics, gas exchange, and radiographic findings prior to implantation, providing a controlled platform to assess graft suitability.^11^, ^12^, ^13^

Although previous meta-analyses have evaluated outcomes comparing DBD and DCD donors, as well as different EVLP protocols,^14^, ^15^, ^16^, ^17^ there is no pooled statistical analysis addressing the role of EVLP in DCD lung transplantation. Given the growing interest in this approach to expand the donor pool, this systematic review and meta-analysis aimed to determine whether the use of EVLP in DCD lungs is associated with improved clinical outcomes compared to direct transplantation without EVLP.

## Methods

This systematic review and meta-analysis followed the Preferred Reporting Items for Systematic Reviews and Meta-Analyses (PRISMA) guidelines^18^ and the Cochrane Handbook for Systematic Reviews of Interventions.^19^ A completed PRISMA 2020 checklist is provided in Supplementary Table 1. The protocol was registered in the International Prospective Register of Systematic Reviews (PROSPERO; CRD420251137577).

### Search strategy

A comprehensive literature search was performed on MEDLINE, EMBASE, and Cochrane Library databases from inception through July 05, 2025, using the following terms: "Lung Transplantation," "lung transplant," "lung graft," "EVLP," “DCD,” "donation after cardiac death." Reverse snowballing technique was performed by searching for eligible studies through references from all included studies. The complete search strategy is presented in Supplementary Table 2.

### Study selection

Two authors (B.S. and B.C.) independently screened the records after removing duplicates. Any disagreements were resolved through discussion with a third author (E.A.). Titles and abstracts were reviewed against predefined inclusion and exclusion criteria.

### Eligibility criteria

Inclusion in this meta-analysis was restricted to studies that met all the following criteria: (1) randomized controlled trials or observational studies; (2) enrollment of adult patients undergoing lung transplantation comparing recipients of lungs from DCD with or without the use of EVLP; (3) reporting at least one outcome of interest; and (4) publication in English. Studies were excluded if they focused on transplantation after brain death, involved animal models, were case reports, abstracts, letters, or editorials, did not report outcomes of interest, lacked a control group, or included overlapping populations.

### Quality assessment and publication bias

Two authors (B.S. and B.C.) independently assessed the quality of included studies using the Cochrane Collaboration tool for assessing the risk of bias in non-randomized studies.^20^ Disagreements were resolved by consensus. Publication bias was assessed only by visual inspection of the funnel plot, as formal statistical tests such as Egger’s test are recommended only when at least 10 studies are available.

### Data extraction

Data extraction from included studies was performed independently by 2 reviewers (B.S. and I.G.) following the predefined criteria. Any conflicts were resolved by consensus and discussion with the senior author (E.A.). The extracted variables included study characteristics (publication year, time frame, country, sample size, and reported outcomes) as well as patient demographics (age, sex, indication, and lung allocation score).

### Outcomes

The main outcome was the occurrence of grade 3 PGD, defined according to the ISHLT criteria.^21^ Additional outcomes included: (1) intensive care unit (ICU) length of stay (LOS) and hospital LOS, both expressed in days; and (2) short- and mid-term survival (90-day and 6-month), and (3) postoperative complications including pneumonia and acute rejection.

### Statistical analysis

Risk ratios (RRs) with 95% confidence intervals (CIs) were calculated for binary outcomes, and mean differences (MDs) with 95% CIs were calculated for continuous outcomes. When appropriate, medians were converted to means and standard deviations using the Luo and Wang method.^22^, ^23^ Heterogeneity was assessed using the Cochrane *Q* test and the *I*² statistic. A *p*-value <0.10 was considered indicative of significant heterogeneity, which was classified as low (I² < 25%), moderate (*I*² = 25%-50%), or high (*I*² > 50%). Statistical significance between groups was set at *p* < 0.05. All pooled analyses were conducted using random-effects models with Restricted Maximum Likelihood estimation to account for between-study variability. The Cochrane Handbook for Systematic Reviews of Interventions was used for data handling and conversion.^19^

Statistical analyses were restricted to outcomes reported in at least 3 studies. Outcomes that were assessed in fewer than 3 studies were not pooled quantitatively; instead, they were summarized narratively and critically appraised.

To assess the robustness of the findings, a leave-one-out sensitivity analysis was performed for the main outcome and for the outcomes with significant heterogeneity. Publication bias was also assessed for the main outcome. All statistical analyses were performed using R software (R Foundation for Statistical Computing, Vienna, Austria), version 4.5.0.^24^

## Results

### Study characteristics

**Figure 1:**
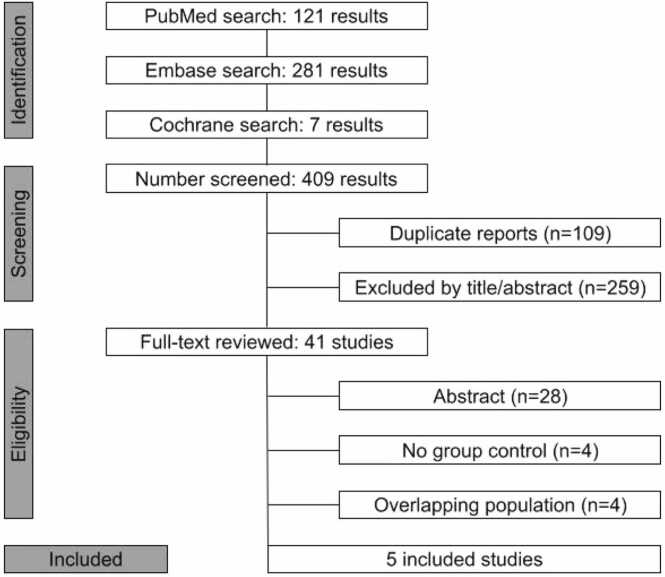
Preferred Reporting Items for Systematic Reviews and Meta-Analysis (PRISMA) flow diagram of study screening and selection.

Figure 1 shows the PRISMA flow diagram of the study selection process. From 409 records initially identified, 5 observational studies met the eligibility criteria for inclusion in the final analysis.^25^, ^26^, ^27^, ^28^, ^29^ The included studies were published between 2015 and 2025, encompassing 654 patients, of whom 304 (46.5%) received DCD lungs treated with EVLP. The mean age ranged from 50 to 63 years, and female representation varied from 25% to 57%. The most frequent indications for transplantation were chronic obstructive pulmonary disease, cystic fibrosis, and restrictive lung disease. Donors were relatively young, with mean age ranging between 32 and 48 years, with balanced sex distribution and preserved oxygenation capacity (PaO₂/FiO₂ ratios >350 mmHg). The baseline characteristics of the included studies are summarized in Table 1, while detailed characteristics of recipients and donors are presented in Supplementary Table 3.

**Table 1:** Baseline Characteristics of the Included Studies

Author, year	Time frame	Study design	Country	Sample size EVLP/No EVLP, *n* (%)	Age (years) EVLP/No EVLP, mean or median	Female EVLP/ No EVLP, *n* (%)	LASEVLP/ No EVLP, mean or median	PaO_2_/FiO_2_ EVLP/ No EVLP, mean or median
Gouchoe et al, 2025^25^	2018-2024	Retrospective	USA	217(50)/217(50)	63/63*	94 (43)/90 (42)	NR/NR	418/411^$^
Kashem et al, 2024^26^	2016-2022	Retrospective^#^	USA and Europe	35(26)/100(74)	57.6/58.1*	15 (43)/39 (39)	43/44*	410/422*
Luc et al, 2017^27^	2011-2015	Retrospective	Canada	7(64)/4(36)	52/58*	4 (57)/1 (25)	NR/NR	367/392*
Machuca et al, 2015^28^	2007-2013	Retrospective	Canada	28(51)/27(49)	52/50*	12 (43)/12 (45)	NR/NR	380/429
Mallea et al, 2022^29^	2015-2019	Retrospective	USA	17(89)/2(11)	62/57^$^	9 (53)/0 (0)	35.35/ 33.21^$^	374/404^$^

Abbreviations: EVLP, ex vivo lung perfusion; LAS, Lung Allocation Score; NR, Not reported.

*mean; $ median; #the study has some prospective collections.

**Table 2:** Summary of Outcomes

Outcome	Number of studies	Effect estimate, random model (95%CI; I² *p*-value)
Grade 3 primary graft dysfunction	4	RR 1.29; 95%CI 0.97 to 1.71; *p* = 0.08; *I*² = 12.63%
Intensive care unit LOS	4	MD −2.46 days; 95%CI −5.57 to 0.65; *p* = 0.12; *I*² = 0%
Hospital LOS	5	MD 0.88 days; 95%CI −7.38 to 9.15; *p* = 0.83; *I*² = 72.2%

Abbreviation: LOS, length of stay.

## Outcomes

### Grade 3 PGD

**Figure 2:**
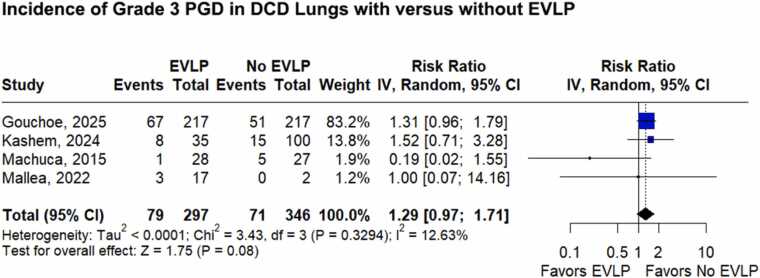
Forest plot comparing grade 3 PGD incidence in recipients of DCD lungs treated with versus without EVLP. DCD, donation after circulatory death; EVLP, ex vivo lung perfusion; PGD, primary graft dysfunction.

Figure 2 presents the pooled analysis of grade 3 PGD incidence, based on 4 studies.^25^, ^26^, ^27^, ^28^, ^29^ Three studies^26^, ^28^, ^29^ assessed PGD at 72 hours post-transplant, whereas one did not specify the assessment time.^25^ The incidence of grade 3 PGD did not differ significantly between recipients of DCD lungs treated with EVLP and those transplanted without EVLP (RR 1.29; 95%CI 0.97 to 1.71; *p* = 0.08; *I*² = 12.63%; Figure 2). All studies, except Machuca et al,^28^ applied the 2016 ISHLT criteria^21^ for PGD assessment.

### ICU and hospital LOS

**Figure 3:**
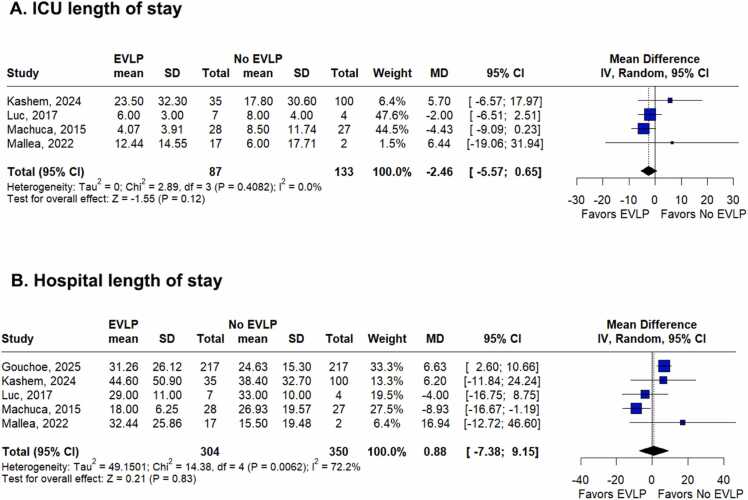
Forest plot comparing **(A)** ICU LOS and **(B)** hospital LOS in recipients of DCD lungs treated with versus without EVLP. CI, confidence interval; EVLP, ex vivo lung perfusion; ICU, intensive care unit; LOS, length of stay; MD, mean difference.

ICU LOS was assessed in 4 studies.^26^, ^27^, ^28^, ^29^ As shown in Figure 3A, it did not differ significantly between recipients of DCD lungs treated with EVLP and those transplanted without EVLP (MD −2.46 days; 95%CI –5.57 to 0.65; *p* = 0.12; *I*² = 0%; Figure 3A).

Regarding hospital LOS, analyzed in 5 studies,^25^, ^26^, ^27^, ^28^, ^29^ Figure 3B shows that there was no significant difference between recipients of DCD lungs treated with and without EVLP (MD 0.88 days; 95%CI −7.38 to 9.15; *p* = 0.83; *I*² = 72.2%; Figure 3B).

### Short-term survival - qualitative analysis

Because survival data were reported heterogeneously across studies, a qualitative synthesis was performed. Overall, short-term survival appeared similar between DCD recipients whose lungs were treated with EVLP and those transplanted without EVLP. Abul Kashem et al^26^ reported comparable 90-day survival (EVLP: 31/35, 88.6% vs no-EVLP: 93/100, 93%), and Mallea et al^29^ similarly observed no difference between groups, reporting no deaths in either group at 90 days (EVLP: 17/17, 100% vs no-EVLP: 2/2, 100%). At 6 months, survival also did not differ substantially, with Luc et al^27^ reporting identical outcomes (EVLP: 7/7, 100% vs no-EVLP: 4/4, 100%) and Mallea et al^28^ again demonstrating comparable results (EVLP: 16/17, 94.1% vs no-EVLP: 1/1, 100%). Consistent with these findings, Machuca et al^28^ observed no significant difference in survival curves between DCD EVLP and DCD without EVLP (*p* = 0.68), with 6-month survival of 86% vs 92% and 1-year survival of 77% vs 92%, respectively.

### Qualitative synthesis of pulmonary complications

Although the absolute number of pneumonia cases was higher in the no-EVLP group, the proportions were similar between groups in the study by Kashem et al^26^ (EVLP: 10/35, 28.6% vs. no-EVLP: 28/100, 28%). In contrast, Mallea et al^29^ reported pneumonia in 1 of 2 patients (50%) in the no-EVLP group compared with 2 of 17 (11.8%) in the EVLP group.

With respect to acute rejection, Gouchoe et al^25^ reported a higher number of cases requiring immunosuppressive treatment in the EVLP group (19/217, 8.8% vs 16/217, 7.4%). Both groups presented the same frequency of untreated acute rejection, with 2 cases each. Mallea et al^29^ also found a higher incidence of acute rejection among EVLP recipients; however, interpretation is limited by the difference in sample size between groups (no-EVLP: 1/2, 50% vs EVLP: 3/17, 17.6%).

### Sensitivity analysis

A leave-one-out sensitivity analysis was performed for the primary outcome (Supplementary Figure 1). Sequential exclusion of individual studies did not materially alter the direction of the effect for grade 3 PGD. Heterogeneity varied depending on the study removed; notably, omission of Machuca et al^28^ eliminated heterogeneity (*I*² = 0%) and produced a p-value at the threshold of statistical significance (*p* = 0.05), indicating a borderline trend toward a higher incidence of PGD-3 in the EVLP group.

Given the substantial heterogeneity observed for hospital LOS, a separate leave-one-out analysis was conducted (Supplementary Figure 2). Excluding Machuca et al^28^ again reduced heterogeneity to 0% and resulted in a statistically significant pooled estimate (*p* =0.047; *I*² = 0%).

Visual inspection of the funnel plot showed no evidence of asymmetry, suggesting no major publication bias (Supplementary Figure 3). Egger’s regression test was not performed because fewer than ten studies were available.

### Risk of bias assessment

**Figure 4:**
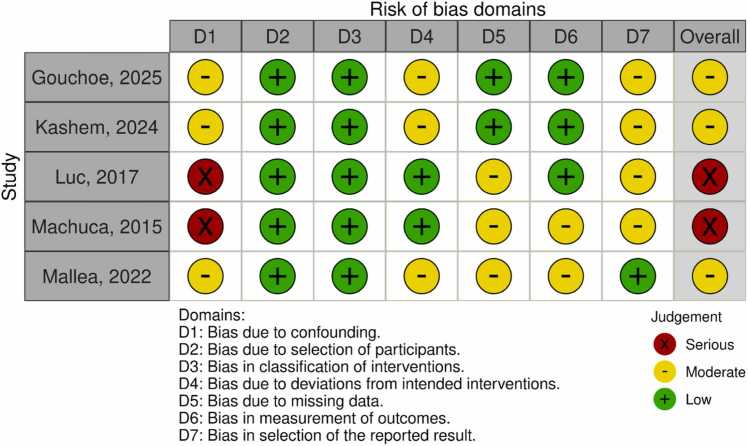
Risk of bias summary for observational studies (RoBINS-I).

Figure 4 summarizes the risk of bias assessment. Across the included studies, 3 were considered to have moderate risk of bias.^25^, ^26^, ^29^ Luc et al^27^ and Machuca et al^28^ were considered to have a serious risk of bias due to confounding.

## Discussion

In this systematic review and meta-analysis of 5 studies including 654 patients, we compared outcomes of lung transplantation from DCD donors with and without the use of EVLP as a graft optimization strategy. Our main findings were as follows: (1) the incidence of grade 3 PGD was comparable between groups; (2) ICU and hospital LOS were comparable between groups; and (3) survival, pneumonia, and acute rejection were assessed qualitatively, showing comparable short- and mid-term survival rates and similar rates of pneumonia and acute rejection between EVLP and no-EVLP recipients.

EVLP has not demonstrated superiority over static cold storage in standard-criteria donors; however, in the specific context of DCD, where the risk of prolonged warm ischemia is greater, it provides a valuable platform for physiological assessment and potential reconditioning before transplantation. In our analysis, the incidence of grade 3 PGD did not differ significantly between groups. Such a finding underscores the limited statistical power and potential heterogeneity in patient selection across available studies rather than a true biological signal of harm. Importantly, our results align with large registry and multicenter data showing that EVLP is not independently associated with increased PGD when adjusted for donor quality, ischemic time, and graft performance.^29^, ^30^, ^31^, ^32^

Moreover, it is important to highlight that, beyond its role in donor selection, EVLP has also facilitated an increase in single-lung transplants, often unacceptable without physiologic assessment, while also being associated with improvements in PaO₂/FiO₂ ratios and hemodynamic and mechanical stability in many patients.^11^, ^29^, ^33^ Importantly, it allows the evaluation of biomarkers such as cytokines and damage-associated molecular patterns, which are associated with increased endothelial permeability and the development of grade 3 PGD, with potential prognostic value for adverse outcomes.^14^

Furthermore, EVLP provides a therapeutic window, as normothermic perfusion supports aerobic metabolism, facilitates clearance of airway secretions, and enables delivery of targeted therapies, with the potential to modulate graft biology at the molecular level.^12^, ^14^, ^27^, ^34^ Review studies further suggest that EVLP exerts intrinsic anti-inflammatory effects by downregulating pro-inflammatory pathways and promoting anti-inflammatory responses, thereby reducing the release of damage-associated molecular patterns and lowering levels of cytokines such as IL-6, IL-1β, IL-18, and TNF-α.^34^ Benazzo et al^35^ reported that EVLP recipients had a higher incidence of PGD-3 at ICU admission compared with conventional transplants. However, in patients who developed PGD-3 after EVLP, the dysfunction resolved rapidly, typically within 24 hours, and these recipients required shorter durations of mechanical ventilation, ICU stay, and overall hospitalization than patients who developed PGD-3 after conventional transplantation.

In this context, EVLP has been primarily concentrated on higher-risk grafts, especially those from DCD donors. Growing evidence, however, indicates that carefully selected DCD lungs can be safely transplanted without EVLP, achieving outcomes comparable to DBD lungs and to direct transplantation. Consequently, many centers no longer perfuse all DCD lungs, instead reserving EVLP for cases with uncertain graft quality, such as compromised oxygenation, prolonged agonal time, pulmonary edema on imaging, or other donor-related concerns. This shift also reflects recognition that EVLP may occasionally lead to the discard of lungs that would have been suitable for transplant. Overall, current evidence supports a selective EVLP strategy, allowing well-preserved DCD lungs that meet standard criteria to proceed directly to transplantation.^28^, ^33^, ^36^

While the impact of EVLP on early graft function appears neutral, contradictory findings in literature warrant caution against its indiscriminate use. Benazzo et al^35^ reported that EVLP may improve mild to moderate pulmonary edema, which is related to capillary fluid extravasation into the lung and evidenced by decreasing albumin levels in the perfusate. Such capillary leakage contributes to edema and increased lung weight, which is associated with worse prognosis and often leads to organs being declined for transplantation.^37^

Regarding ICU and hospital LOS, qualitative assessment showed no significant differences between groups, though these findings should be interpreted cautiously given the substantial heterogeneity in hospital LOS. Sensitivity analyses revealed that the pooled estimate was strongly influenced by individual studies, particularly Machuca et al,^28^ suggesting that variability in hospital LOS likely reflects differences in recipient severity within DCD cohorts. In the Machuca study,^28^ EVLP was performed only when recipients were clinically stable enough to tolerate prolonged evaluation, whereas patients transplanted without EVLP were often more urgent or hemodynamically unstable and therefore ineligible for EVLP assessment. Additionally, preservation time in the no-EVLP group was approximately 3 hours shorter, indicating that these recipients underwent more immediate transplantation. Together, these factors suggest that heterogeneity in hospital LOS is likely driven by recipient-level severity rather than any intrinsic effect of EVLP on postoperative recovery, reinforcing the concept that EVLP functions primarily as a selection and optimization tool rather than a determinant of postoperative morbidity.

Short- and mild-term survival outcomes, also qualitatively analyzed, showed no apparent difference between EVLP and non-EVLP groups at both 90 days and 6 months post transplantaiton. These findings are consistent with previous studies and registry analyses demonstrating comparable early survival after DCD lung transplantation, regardless of EVLP use.^30^, ^31^, ^32^ Similar to our observations, multicenter cohorts have reported equivalent short-term outcomes, even in the presence of longer ischemic times or extended-criteria donor utilization in the EVLP group.^30^ Although some reports described a higher frequency of postoperative complications such as pneumonia, airway dehiscence, or acute rejection among EVLP recipients, these events did not appear to influence early survival in our qualitative synthesis. Collectively, the current evidence supports the safety of EVLP as a graft optimization strategy for DCD lungs, without compromising early post-transplant survival.

An essential aspect in interpreting these results is the potential influence of selection and treatment allocation bias inherent to EVLP use. Although all included studies reported normal PaO₂/FiO₂ ratios prior to transplantation, EVLP is often applied to grafts perceived as marginal or high-risk based on factors such as oxygenation levels, radiographic abnormalities, extended ischemic times, donor age, or a history of smoking.^25^, ^26^, ^27^, ^28^, ^29^ Consequently, donor lungs assigned to EVLP may differ systematically from those transplanted directly, not only in baseline characteristics but also in perioperative management strategies. Previous reports confirm that EVLP is primarily used as a reconditioning and evaluation tool for grafts that would otherwise be declined for transplantation.^11^, ^12^ Therefore, the slightly higher PGD incidence observed in some cohorts likely reflects these underlying donor and procedural differences rather than any intrinsic adverse effect of EVLP. Collectively, our findings support the role of EVLP in safely expanding the donor pool and facilitating the use of DCD lungs without compromising early outcomes, ultimately contributing to increased graft utilization and reduced waitlist mortality.^1^, ^2^, ^3^, ^4^

Looking ahead, while our findings support the safety of EVLP for DCD lung transplantation without demonstrating clear clinical superiority, they also highlight opportunities for refinement. Future research should focus on multicenter randomized trials stratified by donor type and recipient risk profile, while incorporating inflammatory biomarkers obtained during EVLP as potential predictors of clinical outcomes.^14^ In parallel, the development of intrapulmonary therapies administered during EVLP, including anti-inflammatory and immunomodulatory agents, could transform this platform into an active reconditioning tool.^14^, ^27^, ^36^ Widespread adoption of such strategies could ultimately redefine the use of DCD lungs in transplantation, balancing early risks with the long-term benefit of safely expanding the donor pool.

This study has limitations. First, all included studies were observational, with inherent susceptibility to selection bias, treatment allocation bias, and confounding. Second, EVLP protocols, devices, and acceptance criteria varied among centers, introducing clinical heterogeneity. Third, many studies on EVLP are single-arm cohorts without a comparative group, and excluding these studies in our review limits the breadth of clinical experience considered. Fourth, our meta-analysis included fewer than 650 patients across only 5 studies, limiting statistical power and precluding a reliable assessment of publication bias. Nevertheless, while our results should be interpreted with caution, they are consistent with existing evidence and provide important hypothesis-generating insights for future prospective multicenter studies and randomized trials.

## Conclusion

EVLP in DCD lung transplantation was not associated with significant differences in grade 3 PGD, ICU or hospital LOS, or short- to mid-term outcomes compared with direct transplantation. Given its time and resource demands, EVLP may not be necessary for all DCD grafts; however, it remains particularly valuable for evaluating uncertain-quality lungs, where its selective use can help ensure graft safety.

## Financial support

TC was funded by the Deutsche Forschungsgemeinschaft (DFG, German Research Foundation) Clinician Scientist Program OrganAge funding number 413668513, by the Deutsche Herzstiftung (DHS, German Heart Foundation) funding number S/03/23, and by the Interdisciplinary Center of Clinical Research of the Medical Faculty Jena.

## Data Availability Statement

The data underlying this article are available in the article and in its online Supplementary Material.

## Disclosure statement

None.

## Conflicts of Interest statement

The authors declare that they have no known competing financial interests or personal relationships that could have appeared to influence the work reported in this paper.

## Footnotes

Appendix A Supplementary data associated with this article can be found in the online version at doi:10.1016/j.jhlto.2026.100480.
